# From Accelerometer to Cognition: Hand Motion Can Reflect Effects of Cardiac Coherence on Cognitive Flexibility

**DOI:** 10.3390/s25092942

**Published:** 2025-05-07

**Authors:** Alix Bouni, Laurent M. Arsac, Olivier Chevalerias, Véronique Deschodt-Arsac

**Affiliations:** 1 University of Bordeaux, CNRS, Laboratoire IMS, UMR 5218, 33405 Talence, France; a.bouni@catie.fr (A.B.); veronique.arsac@u-bordeaux.fr (V.D.-A.); 2 Centre Aquitain des Technologies de l’Information et Electroniques, 33400 Talence, France; o.chevalerias@catie.fr

**Keywords:** human monitoring, cognition, movement, multifractality

## Abstract

Hand displacements during task-directed movements are not random, but exhibit fractal behavior. Wearable sensing e.g., accelerometer-derived hand movement fluctuations, could add a significant contribution to cognitive and behavioral sciences, by accounting for fractal dynamics. In particular, multifractal testing of fluctuation time series has been shown to reflect the adaptive use of cognition, i.e., cognitive flexibility. This important property might be enhanced by an improved mental state. Here, an experimental group (16 participants, 3 females) practiced 5 min cardiac coherence (CC) prior to a cognitive flexibility task and was compared to a control group (13 participants, 4 females). Accelerometer-derived hand motion was analyzed using multifractal-multiscale detrended fluctuation analysis (MFMS-DFA) during a task involving cognitive flexibility, the Wisconsin Card Sorting Test (WCST). WCST included four phases alternating the use of cards with original shapes or animal pictures developed for children in previous research. Hand behavioral time series derived from the wearable accelerometer effectively exhibited nonlinear multifractality as shown using linearized surrogates testing. Multifractal-multiscale metrics revealed significant effects of pre-task CC practice, specifically during WCST shape condition where CC participants showed lower multifractal degree despite similar performances (perseverative errors). We conclude that multifractal-multiscale testing of accelerometer-derived hand motion could make a significant contribution to interpreting changes in cognitive flexibility.

## 1. Introduction

The rapid development of small-sized sensors allows wearable sensing technology to make a significant contribution to advances in several scientific fields. In this regard, accelerometers offer a great opportunity for behavioral monitoring in humans. Specifically, with their capacity to capture fluctuations in hand motions with limited intrusiveness and high accuracy, accelerometer-derived signals might add significant value for behavioral and cognitive sciences.

During task-directed actions, fluctuations in hand movements do not resemble random white noise, but exhibit a particular temporal, so-called fractal structure; this has become an attractive phenomenon to account for cognition [1,2,3,4,5]. A number of cognitive and physiological systems indeed exhibit fractal coordination, which is reflected in self-similarity in the temporal structure of the output signal [6,7]. In this context, previous researchers have explored the relationship between hand motion and cognition. They showed that hand movement behavior closely matched changes in cognitive functioning [1,4,8,9,10,11]. This interesting framework could find further developments by exploiting the capacity of non-intrusive wearable sensors to capture hand behavior.

The cognitive system is designed to generate and maintain goal-directed actions. The ability to shift attentional focus, switch from one rule to another, and adjust behavior reflects cognitive flexibility [12]. This is an important ability relying on the coordination of perception, cognition, and action and is an interaction-dominant process spreading across several time scales, providing humans with the essential property of being able to cope with ever-changing contexts. A formal link between accelerometer-derived hand movements and cognitive flexibility has not been established to date. Yet, appealing approaches have shown that hand behavior during task-directed actions exhibits a particular form of fractal behavior known as nonlinear multifractality [2,4]. Thus, we should expect nonlinear multifractality in accelerometer signals, and changes in multifractal metrics in case of an alteration in the cognitive system. Disease and aging are known candidates that alter mental flexibility [13]. One first step to endorse a framework linking accelerometer-derived signals to cognitive flexibility could be to involve healthy people and introduce a pre-task stimulation able to affect cognitive functioning during the task. Simple practices could be implemented to interfere with the cognitive system by affecting the brain. Among them, cardiac coherence (CC) consists of achieving resonance between the two main oscillators controlling the heart rhythm to generate a bottom-up flux through the central autonomic network [14]. CC is generally achieved by controlling a slow breathing rate at 0.1 Hz to synchronize the respiratory-driven parasympathetic frequency with the 0.1 Hz sympathetic frequency [15]. Such stimulation has been shown to improve the mental state, with reduced anxiety and improved attentional resources [16]. We are not aware of previous investigations tracking CC effects on cognition in multifractal behavior during task-directed movements.

Multifractal features in the output signal of cognitive and physiological systems have been captured by using different methods, among which, detrended fluctuation analysis (DFA) has made a significant contribution [17]. DFA quantifies the fluctuation sizes of a signal over a range of observational scales by computing a scaling exponent [18]. Fluctuations result from the superposition of the interaction of several system components. If these components act independently, uncorrelated fluctuations resembling white noise are expected and the DFA exponent equals 0.5. By contrast, if the interaction spreads across large structural and temporal scales, the DFA exponent departs from 0.5 to reach e.g., 1.0 in cases of pure self-similarity—so-called fractal coordination [6,19]. On its side, multifractality accounts for the presence of superimposed fractal processes, which is revealed by using a set of q-moments applied to the fluctuation function [3,20]. A finer analysis has been gained by introducing a multiscale dimension based on experimental observations that several systems, namely the control of heart rhythm [20], upright standing posture [21], and movement [22], actually exhibit multifractal spectrum whose size varies with the time scales at which they are observed. The fact that multifractality is more or less marked at certain time scales is critical information that could help to make the link with underlying mechanisms. For instance, when the movement system is involved in postural control, a crossover that distinguishes between fast and slow processes indicates scale-specific power-law behavior, and the appearance and disappearance of crossovers reveal that non-Gaussian cascade dynamics extend from long into short time scale [21]. Recent refinements of a multifractal-multiscale application of DFA, MFMS-DFA, are appealing [20,23]. The method allows a detailed analysis of behavioral adaptations by providing a set of q,s-dependent values of the DFA exponent: *α*(q,s).

Here, we used a wearable device attached to the hand of young and healthy participants in order to capture hand acceleration series, as the main characteristic of behavior, while they were sorting cards. In particular, the Wisconsin Card Sorting Test [24] was employed as a cognitive constraint to stimulate mental flexibility due to the fact that participants must infer the sorting rule from experimenter feedback and the rule often changes during sorting. A detailed analysis of the temporal structure of behavior was obtained by using MFMS-DFA, which provides critical metrics of a multifractal structure, a set of *α*(*q*,*s*). By using CC prior to the WCST performance, we aimed to test if our approach from accelerometer to cognition is sensitive enough to detect subtle changes in the adaptive use of cognition.

## 2. Materials and Methods

### 2.1. Participants

Twenty-nine young, healthy, and physically active participants (20.2 ± 3.0 years, 7 women; 11.7 ± 6.8 h of sports per week) provided written informed consent to participate in the present study, which was approved by the Institutional Review Board of the Faculte des STAPS. The procedures adhered to ethical recommendations and followed the guidelines of the Declaration of Helsinki. Participants were recruited among sports students at the University of Bordeaux. Eligibility criteria included the absence of cardiovascular disease or uncorrected visual disorders. Participants were instructed to avoid alcohol and caffeinated beverages for 12 h before the session and to refrain from strenuous physical activity the day before the experiment. They were randomly assigned to either an experimental (EXP) or a control (CTRL) group.

### 2.2. Procedure

The protocol consisted of one single session lasting ±45 min. Upon their arrival at the lab, participants had the procedure explained to them. Then, to obtain a baseline condition, they watched an emotionally neutral video. The EXP group stopped watching the video after 8 min to undergo 5 min of cardiac coherence practice (described in Section 2.4), which was the only difference from the CTRL group. The CTRL group watched a 13 min video. Then, both groups performed a card sorting task, the Wisconsin Card Sorting Test (WCST) for 8 min, comprising four successive 2 min phases where card desks changed (Section 2.3.1). Immediately after WCST, the participants answered a NASA-TLX questionnaire aiming to assess their perceived cognitive load.

### 2.3. The Cognitive Task—Card Sorting (WCST)

#### 2.3.1. Task Description

**Figure 1:**
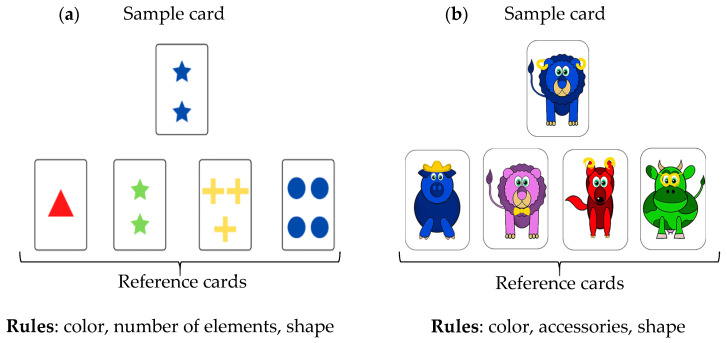
Illustration of the card-sorting task using cards with shapes (triangle, star, cross, disc) on the left panel or animals (pig, lion, fox, cow) on the right panel. Participants were presented with four reference cards (at the bottom of the figure) and were required to grasp a card and place it in front of the appropriate reference pile according to the rule for that round. (**a**) Original WCST; (**b**) WCST with animals.

The Wisconsin Card Sorting Test (WCST) is a cognitive assessment tool designed to evaluate mental flexibility [24]. In this task, the participant grasped a card from a card desk and had to sort it by matching one of the four cards laid out in front of them within reach. The four cards indicate four references according to one of three implicit rules (see below). The rule in effect was not explicitly stated; the participant must deduce it based on verbal feedback provided by the experimenter after each sorting attempt, indicating whether the choice was “correct” or “incorrect”. During the task, the rule changed without the participant being informed. To add task complexity, we modified the pseudo-random rule once the participant had successfully identified it. Specifically, after three consecutive correct trials, the experimenter was informed of a random number of trials between 1 and 6 to determine when the next rule change occurred. We also used two distinct card desks to introduce an alternative set of pictures on the cards (Figure 1): the original WCST cards with shapes and a set of cards with animals that was developed for WCST involving children [1].

The four 2 min phases took place as follows: shapes (phase 1), animals (phase 2), shapes (phase 3), and animals (phase 4). The rules associated with shapes were color, shape, and number of items; the rules associated with animals were color, animal, and type of accessories worn.

#### 2.3.2. Analysis of WCST Performance

WCST performance is often assessed in terms of the number of persistent responses and the number of errors on the first change in rule. Yet, such metrics do not consider the participant’s chance of identifying the new rule on the first or second attempt, which does not directly reflect mental flexibility. Given that participants have three possible sorting options, when the participant makes more than two consecutive errors following a rule change, these errors cannot be attributed to bad luck, but rather to the difficulty in adapting to the new rule. So here, WCST performance was based on counting perseverative errors >2.

### 2.4. Cardiac Coherence and Measurement of Heart Rate Variability

Guided breathing at 0.1 Hz (5 s inspiration/5 s expiration) was assisted by a custom-made handheld device (developed by a start-up, URGO) held in the dominant hand. The device stimulated breathing with synchronized visuo-haptic signals for 5 min. Haptic guidance consisted of vibrations whose frequency gradually increased during the 5 s inspiration phase and decreased during the 5 s expiration phase. Visual guidance included a blue light signal visible on the device, which also varied according to the inspiration and expiration phases [25].

During the experiment, we measured heart rate variability (HRV) using a Z_ECG device, sampled at 1 kHz (https://6tron.io/z_object/z_ecg_1_0_0/, accessed on 30 March 2025) and attached to a Polar belt (Polar Electro Oy, Finland) (the device developed by CATIE is based on Texas Instruments, AFE4960, ECG analog front-end). Rpeak-to-Rpeak detection was performed in Matlab (Matlab R2023a, The Mathworks, Natick, MA, USA) using wavelet processing (Daubechies 4) on the raw ECG data and the findpeaks function to detect and extract the interval between R waves. Heart rate variability (HRV) was explored as RR series inspected for artifacts, occasional ectopic beats (irregularity of heart rhythm involving extra or skipped heartbeats, e.g., extrasystoles and consecutive compensatory pauses). When necessary, ectopic and artifact data were manually replaced with interpolated adjacent values.

To assess the quality of cardiac coherence, we used the P_0.1_ indicator, as suggested in [26], which is the ratio of the HRV power spectral density (PSD) around 0.1 Hz during Cardiac Coherence to the HRV autonomous power distributed between 0.04 and 0.4 Hz during baseline. As CC aims at concentrating the whole autonomous power at 0.1 Hz, the higher the P_0.1_ ratio, the higher the success of CC.

### 2.5. Data Collection

**Figure 2:**
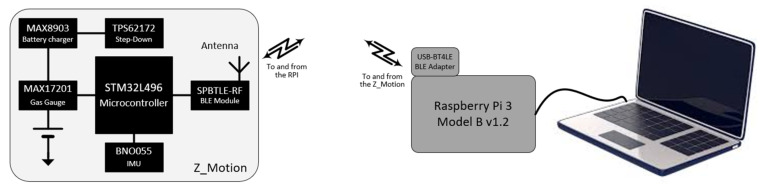
Illustration of the recording system used, including the wearable device Z_motion (left panel). See text for details.

Data were collected with a system developed by the Centre Aquitain des Technologies de l’Information et Electroniques (CATIE, Bordeaux, France). It is based on a Raspberry Pi 3 Model B v1.2 that acts as a control and storage unit (Figure 2). Its role is to ensure proper synchronization of different sensors in use and to collect and store the incoming data during the experiment. A Bluetooth Low Energy link provides an efficient communication solution between the unit and the sensors. The Raspberry Pi is equipped with a USB 2.0 Bluetooth adapter (USB-BT4LE, Plugable, Redmond, WA, USA) to improve the reliability of the wireless link. At system startup, the Raspberry Pi detects the different sensors and pairs with them. When the experiment is ready, a synchronization message is emitted. This resets the time base on the sensors and triggers the acquisition cycle. The signal is digitized and transmitted. At reception on the Raspberry Pi, data are written on a microSD card (SanDisk Ultra16 GB, Milpitas, CA, USA) as a CSV file. For this experiment, the sensor was a Z_Motion (v1.0.0, https://catie-na.fr/produit/z_motion/, accessed on 30 March 2025), a wireless battery-powered motion sensor designed by CATIE. The device includes a microcontroller (STM32L496, STMicroelectronics, Geneva, Switzerland) and a Bluetooth module (SPBTLE-RF, STMicroelectronics, Geneva, Switzerland), a 165 mAh Lithium Polymer rechargeable battery (LP-402025-IS-3, BAK, Shenzen, China) with a charger IC (MAX8903, Analog Devices, Wilmington, DE, USA) and a fuel gauge (MAX17201, Analog Devices, Wilmington, DE, USA), a Step-Down converter (TPS62172, Texas Instruments, Dallas, TX, USA), and a 9-Axis Inertial Measurement Unit (IMU), BNO055 (Bosch Sensortec, Reutlingen, Germany). Only the data from the triaxial 14-bit accelerometer were recorded with a default programmed range of ±4 g and a sampling frequency of 100 Hz. Timestamped data were transmitted to the collection unit in packets to reduce the band occupancy and power consumption. At the end of the experiment, an end message was emitted by the Raspberry Pi. At reception, the sensors interrupted the acquisition process and stopped the transmission. The CSV file was closed, and the microSD card was simply transferred to a PC where analysis could take place with MATLAB or dedicated software.

The Z_Motion device (0.021 kg) was attached with Velcro tape on a fingerless glove to the back of the hand to monitor the movement of the hand sorting the cards.

### 2.6. Multifractal Multiscale DFA

We analyzed the accelerometer-derived hand acceleration series during card sorting using the MFMS-DFA method described and available in [23]. The method is based on DFA, where linear (rather than quadratic or superior order) detrending was used in the present study. Briefly, we calculated the cumulative sum, *y_i_*, of each series and split *y_i_* into *M* maximally overlapped blocks of *n* samples. Then, we detrended each block with a least-squares linear regression and calculated the variance of the residuals in each *k*-th block, *σ*^2^*n*(*k*). The variability function F*_q_*(*n*) is the *q*-th moment of *σ*^2^*n*.

(1) $$\left\{\begin{array}{c} F_{q}(n)={\left(\frac{1}{M}\sum_{k=1}^{M}{\left(\sigma_{n}^{2}\left(k\right)\right)}^{q/2}\right)}^{1/q}for\;q\neq 0\\ F_{q}(n)=e^{\frac{1}{2M}\sum_{K=1}^{M}ln\left(\sigma_{n}^{2}\left(k\right)\right)}\;for\;q=0 \end{array}\right.$$

We calculated F*_q_*(*n*) for 0 ≤ *q* ≤ 5 and 10 ≤ *n* ≤ 350 to capture the main fluctuation characteristics.

**Figure 3:**
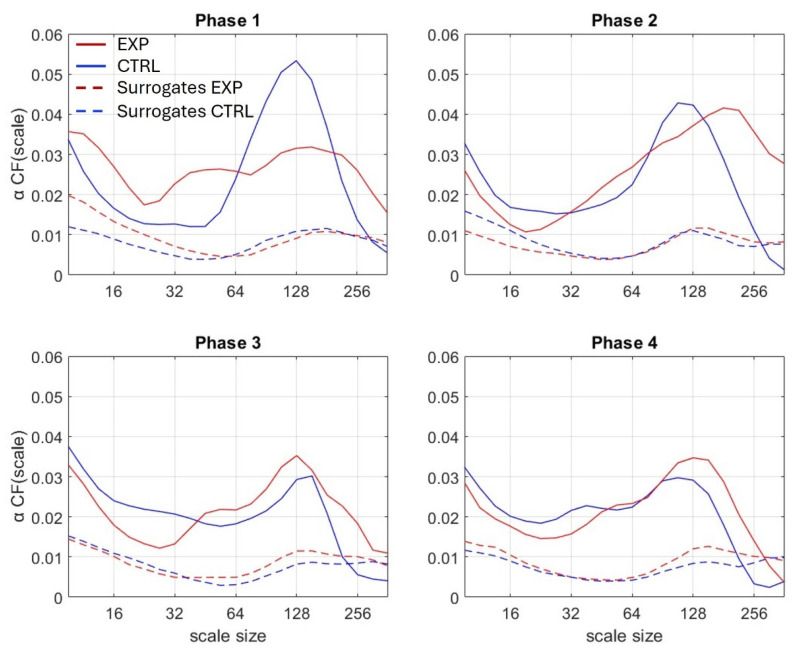
Cumulative function αCF (scale) obtained for each of the 4 phases. Lines represent the median value of EXP (red), and CTRL (blue) group. Corresponding surrogates are represented by dot lines.

To quantify the degree of multifractality at each scale *n*, CF(*n*), we calculated the cumulative function of the *q*-induced squared increments of α(*n*) (Figure 3):

Surrogate data testing allowed the detection of nonlinear features in multifractality. We used the Iterative Amplitude Adjusted Fourier Transform (IAAFT) procedure to generate 40 linearized surrogate series for each original series, and we compared multifractality in original vs. surrogate series (Figure 3).

### 2.7. Statistical Analysis

#### 2.7.1. Performance and NASA-TLX

We assessed subjects’ performance in each phase by analyzing the effect of cardiac coherence on the type of card used, shapes, or animals. To do this, we compared the number of perseverative errors in phases 1 and 3 with those in phases 2 and 4 using a one-tailed Wilcoxon test. In addition, we examined the effect of cardiac coherence for each phase independently using a Wilcoxon test. The NASA-TLX was analyzed by considering the total score reported by each subject. We compared the EXP and CTRL groups using a Wilcoxon test.

#### 2.7.2. MFMS-DFA Metrics

We compared the coefficients α(q,s) for the EXP groups against the CTRL reference using Wilcoxon’s signed-rank test for each q and s. These differences are represented by color maps. These maps visualized the regions of q,s space that showed the most significant differences between CTRL and EXP.

To test nonlinear multifractality in our signals, we compared the cumulative function obtained for each experimental series to the median of 40 IAAFT surrogate series by using a one-tailed Wilcoxon test.

For all statistics, a *p*-value of less than 0.05 was considered statistically significant. Data analysis, statistical analysis, and figure drawing were all performed in Matlab (MatlabR2023a, Matworks, Natick, MA, USA).

## 3. Results

### 3.1. Analysis of Hand Acceleration Series

#### 3.1.1. MFMS-DFA

**Figure 4:**
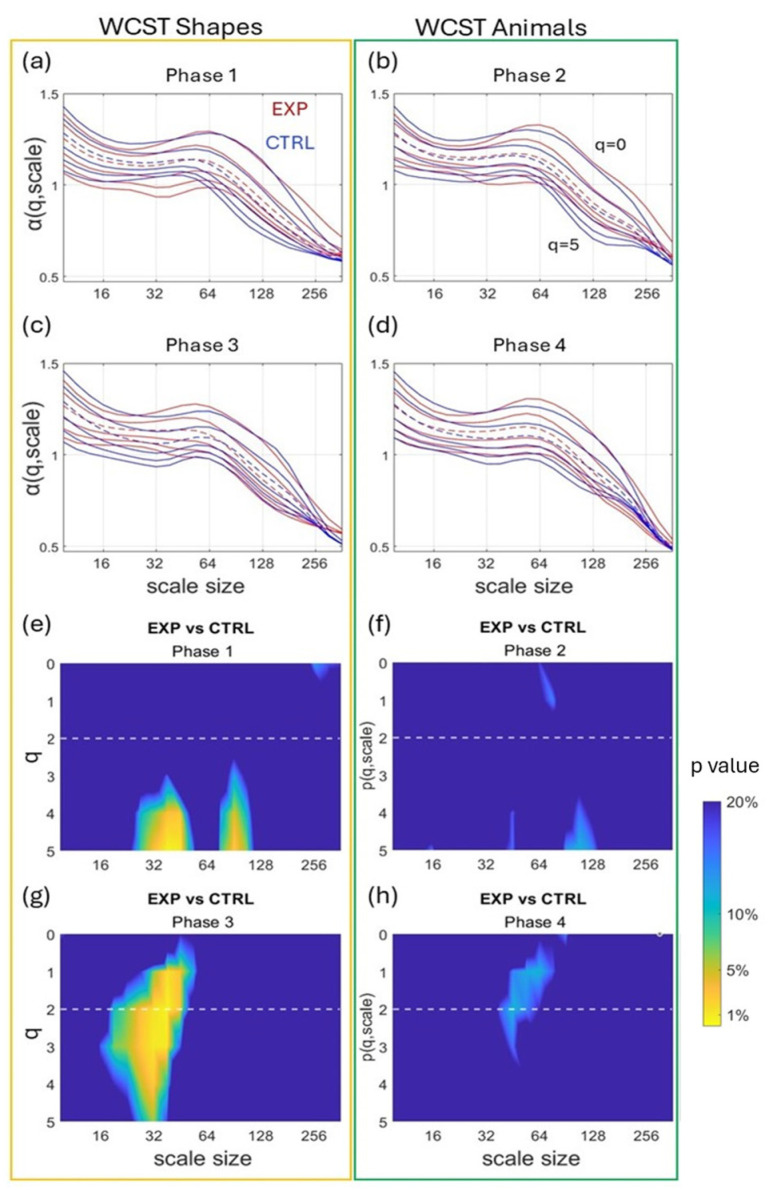
Mean MFMS-DFA coefficients for each group during the four phases (**a**–**d**) of the card-sorting task. α (q, scale) is shown in red for the EXP group and in blue for the CTRL group, for all q between 0 and 5. The dotted lines indicate q = 2 (the moment order of the monofractal DFA). The second part of the figure (**e**–**h**) presents color maps of the statistical significance p, after a Wilcoxon comparison of the MFMS-DFA coefficients of the EXP and CTRL groups during the four phases.

Figure 4 compares α(q,s) coefficients obtained by the MFMS-DFA in EXP and CTRL for each phase. Color maps (Figure 4e–h) show the difference between groups in α(q,s) shown in Figure 4a–d. The difference induced by cardiac coherence is visible as a yellow area in color maps according to the color bar legend. Although cardiac coherence had no effect on α(q,s) values during phase 2 and phase 4 (colormaps are blue (Figure 4f,h), when animals were used, there was a significant effect of CC during phase 1 and phase 3 (bright yellow in mainly scales size 32 for different moments q) that is when participants used cards with shapes (Figure 4e,g). It is worth noting that the effect was reproducible, which can eliminate the possibility of a random result.

#### 3.1.2. Multifractality and Surrogates

We compared the cumulative functions *α*CF of the original series with those of their linearized surrogate series for testing the nonlinearity origin of multifractality. The cumulative function of the original series (area under the curve) was significantly higher than that of the surrogate series (all *p*-values < 0.001).

### 3.2. Perseverative Errors and Cognitive Load

The EXP group showed similar performance when completing sorting with either shape or animal cards. By contrast, the CTRL group made more perseverative errors with shape cards when compared to animal cards (Wilcoxon test, *p* = 0.047).

Individual scores obtained with NASA-TLX indicate a moderate perceived cognitive load after completing the whole task and no difference between EXP (54.2 ± 10.7) and CTRL (59.4 ± 13.2).

### 3.3. Cardiac Coherence Efficiency and Performance

The P_0.1_ index of the EXP group showed good performance during CC (0.9 ± 0.4) when compared to previous results [26]. No correlation between individual P_0.1_ and performance (perseverative errors) was found.

## 4. Discussion

A number of previous studies have shown that hand behavior during task-directed movements is not white noise but exhibits fractal-like behavior [1,4,8]. Here, we show an interesting usage of wearable accelerometers by capturing hand behavior during card sorting to assess cognitive flexibility. Combining accelerometer-derived hand motion with a specific multifractal multiscale analysis, we estimated the adaptive use of cognition in normal and enhanced mental states, obtained by pre-task cardiac coherence practice. As the main finding, cardiac coherence in the EXP group changed the multifractal-multiscale signature of hand behavior (Figure 4) during card sorting with changing rules.

In our conditions, during each 2 min phase of the WCST alternating the use of shape and animal cards, participants had to infer the phase-specific sorting rule. Such a task has been established to stimulate cognitive flexibility, a complex process that has been rooted in nonlinear multifractal behavior [1]. Accordingly, in the present study, the fluctuation series provided by the accelerometer recordings demonstrated greater multifractality than their linearized surrogates (Figure 3), which advocates for nonlinear multifractal behavior, as expected. The consequence of these results is two-fold: first, it provides additional evidence that multifractal analyses closely track cognitive flexibility; second, it introduces wearable accelerometers as reliable tools for such analyses.

A detailed analysis was conducted here using MFMS-DFA to capture scale-dependent multifractal behavior. The analysis of behavioral *α*(q,s) allowed cognitive behaviors in different phases of the WCST to be distinguished when EXP and CTRL groups are compared. In our conditions, the CC group practiced cardiac coherence prior to the task, which is supposed to improve the mental state of the participants. Interestingly, *α*(q,s) were not blind to CC. Color maps in Figure 4 highlight significant differences in *α* values at specific observational scales and for a given range of q-moments. The effect of CC was detected during phase 1 and more markedly during phase 3 of the task around observational scale size 32 (Figure 4g). Phase 1 and phase 3 imposed sorting cards tagged with original shapes, not animal cards that have been employed with children. Our results thus indicate that CC was effective in modifying cognitive flexibility only when shape cards were employed. This result is in agreement with the greater number of perseverative errors noted for the CTRL group only, thus linking MFMS-DFA metrics obtained here with task difficulty.

Shape cards appear to require more abstract processing and active rule recoding, leading to greater involvement of top-down executive control mechanisms. In contrast, animal cards tend to activate rich semantic networks and rely on more automatic, familiar stimulus–response associations, which reduce the demand for active inhibition or flexible rule shifting [12].

This possible interpretation is supported by evidence from neuroimaging studies indicating distinct neural recruitment patterns depending on stimulus type [12]. Tasks involving geometric forms primarily engage the fronto-parietal network [27], particularly regions within the dorsolateral prefrontal cortex, which are responsible for attentional control, rule shifting, and conflict resolution [28]. These regions are known to benefit from targeted cognitive training [29]. Conversely, stimuli with strong semantic or perceptual salience—such as animals—tend to recruit ventral pathways including the orbitofrontal cortex and fusiform gyrus, areas involved in reward processing and object recognition, which are typically less sensitive to cognitive control interventions.

From a cognitive conflict perspective, shape-based tasks may elicit stronger inter-dimensional interference (e.g., shape vs. color), requiring active inhibition of irrelevant features and increased mental flexibility to switch between sorting rules. Animal stimuli, due to their perceptual distinctiveness and semantic familiarity, may instead guide behavior through more automatic, holistic processing strategies, reducing the need for flexible top-down control [30].

Taken together, these observations suggest that CC practice selectively enhances performance in tasks that rely on executive control and abstract cognitive processing. This may explain why changes in multiscale fractal dynamics of hand movements were observed predominantly in the shape condition, where cognitive flexibility demands are objectively higher.

These results could indicate two important aspects of using accelerometer and MFMS-DFA in combination. First, the approach has obvious sensitivity in assessing changes in cognitive flexibility, since the effect of CC has been repeatedly detected during phases 1 and 3, both using shape cards, and the absence of CC effect has been repeatedly detected during phases 2 and 4, both using animal cards, which highlights good reliability. Second, the approach is also qualitative enough to highlight that using shape or animal cards does not engage similar cognitive coordination, or finally, does not have a similar interaction with mental state. As the present study was not designed to provide further interpretations about using shape or animal cards during WCST, our results provide only a first, but important, methodological step. It should be added that monitoring human hand behavior using accelerometers benefits from continuous research capable of providing integration in small rings combined with activity recognition algorithms [31]. The convergence of these technologies with analytical methods such as MFMS-DFA, presented here, opens up promising prospects for identifying, e.g., when an individual arrives at the workplace, then triggering specific sensors that assess their mental state, and possibly suggesting that the individual takes time for remediation. 

The present study is not without limitations. First, the small sample size of our population and the fact that they are all young and physically active restricts the generalization of our results. Second, the placement of an accelerometer, although light, on the hand might affect the movement during the task. However, a similar procedure has already been used with an Apple watch (0.029 kg) attached to the hand, and no discomfort was mentioned [32]. If the device should still influence movement, it should be noted that in the present study, the two groups compared (CTLR and EXP) used the device under similar conditions. Third, although the effect of CC on some aspects of cognitive flexibility was clearly revealed by the present metrics, the time scales at which the effect was dominant can only be explained with additional explorations.

## 5. Conclusions

Combining the use of wearable sensors with multifractal-multiscale approaches to behavior can make a significant contribution to neurosciences and cognitive psychology. We used ‘from accelerometer to cognition’ in the title of the present work and went beyond the slogan to suggest an interesting framework involving wearable devices to monitor normal, degraded, and diseased human behavior.

## Data Availability

Data are available from the corresponding author upon reasonable request.

## Abbreviations

The following abbreviations are used in this manuscript:

WCST	Wisconsin Card Sorting Test
MFMS-DFA	Multifractal-multiscale detrended fluctuation analysis
CC	Cardiac coherence
EXP	Experimental group
CTRL	Control group
IAAFT	Iterative Amplitude Adjusted Fourier Transform
